# Detection of esophageal and glandular stomach calcification in cow (*Bos taurus*)

**DOI:** 10.14202/vetworld.2020.1153-1158

**Published:** 2020-06-19

**Authors:** Esraa Abdul Khaliq Zegyer, Basim Abdullah Al Khuzaee, Ahmed Mahdi Al Badri

**Affiliations:** 1 Department of Pathological Analyses, College of Science, University of Wasit, Wasit, Iraq; 2 Department of Biology, College of Science, University of Al Muthanna, Al Muthanna, Iraq; 3 Department of Biology, College of Science, University of Wasit, Wasit, Iraq

**Keywords:** abomasum, alizarin red, calcification, esophagus, immunohistochemistry, von Kossa

## Abstract

**Aim:**

The aims of this study were first to estimate calcification in the esophagus and abomasum of cows and second to quantify its appearance with increasing age using histopathological and immunohistochemical techniques.

**Materials and Methods:**

Esophageal and abomasal samples from 24 healthy cows (*Bos taurus*) were collected. Hematoxylin and eosin, alizarin red, and von Kossa stains were used for histopathological analysis. Histopathological changes were confirmed with immunohistochemical staining, followed by digital image analysis.

**Results:**

Histological findings revealed the esophagus and abomasum wall comprised four fundamental layers, the mucosa, submucosa, muscularis, and serosa or adventitia. At 1 year old, calcification was beginning to appear as fine diffused points in mucosa, submucosa, and muscularis of both esophagus and abomasum, appearing as small spots at 2 years old. With advancing age in all animals, this calcification began to appear as medium spots spread throughout all wall layers of these organs at 3 years old. By 4 years old, calcification had evolved into large dark foci spread substantially throughout the tunica submucosa and tunica muscularis. Immunohistochemical results exhibited positive immunoreaction to calcium salts in the esophagus and abomasum layers in all animals, which increased with age.

**Conclusion:**

The current study concluded that calcification is a pathological event appearing spontaneously in various types of soft tissue, significantly increasing with age, either because of hypercalcemia and hyperphosphatemia or secondary to other diseases.

## Introduction

Beef and dairy cattle are the main production branches which make up the economic sector of animal husbandry. The previous studies exist surrounding beef and dairy cattle and their role in helping the development of animal agriculture in the field [1]. The gastrointestinal tract (GIT) is one of the heaviest and largest organ systems of most animals. In cows, it comprises the oral cavity, esophagus, forestomachs, abomasum (upper GIT), and the intestines, rectum, and anus (lower GIT). In healthy cattle, these organs occupy more than half of the abdominal cavity and a small space in the peritoneal cavity [2]. GIT diseases in cattle are numerous and pose a significant clinical problem. Upper and lower GIT disorders in adult ruminants can result from a variety of causes, the most common being inflammatory, dietary, and mechanical reasons [3].

Calcification is an uncommon feature of untreated gastrointestinal stromal tumors (GISTs) [4]. Several studies have indicated that the putative cause of initial calcified GISTs is dystrophic calcification, wherein calcium salts precipitate in deteriorating tissues, such as sites of hemorrhage, necrosis, and scarring [5]. Other reports have revealed widespread calcification in a range of soft-tissue organs, including the lungs, brain, kidneys, tear glands, myocardium [6], heart valves [7], and pancreas [8]. Extensive calcifications are extremely rare in the majority of gastrointestinal tumors. However, heavy extraluminal calcified masses have been reported in the stomach, and the first case of stomach GIST was discovered with an intraluminal growth pattern through computed tomography [9].

Thereafter, a few reports started to put forward limited conclusions regarding cases of calcification, including pattern, clinical presentation, and risk of heavy calcification in GIT tumors [10]. The occurrence and diffusion pattern of calcification in the esophagus and GIT have previously been diagnosed and described generally only by radiological detection [11].

Therefore, the aims of this study were first to estimate calcification in the esophagus and abomasum of cows and second to quantify its appearance with increasing age using histopathological and immunohistochemical techniques.

## Materials and Methods

### Ethical approval

This research was carried out under the supervision and control of Wasit University Research Council policy and received approval from the College of Science/Department of Pathological Analyses/Department of Biology. The samples were collected from the slaughtered animals of the slaughterhouse.

### Sample collection

Samples of the esophagus and glandular stomach (abomasum) (48 total) were collected from 24 healthy cows (*Bos taurus*) slaughtered in Wasit abattoir, Iraq in March 2018. All experimental animals were confirmed free of any renal, parathyroid, or other neoplastic diseases at the time of necropsy. Samples were divided into four groups based on the age of animals (1, 2, 3, and 4 years). Age was determined based on the teeth equation [7]. Each group contained 12 samples (6 esophagus and 6 abomasum), no macroscopic lesions were found in any sample, and specimens were considered clinically healthy. The esophagus and abomasum were dissected, and specimens were taken from the cervical part of the esophagus and a random location in the abomasum. All collected specimens were conserved immediately in 10% formalin for 72 h then transferred for histology. For histopathological analysis of the general structure of organs, hematoxylin and eosin (H&E) stain was used [12], while alizarin red and von Kossa stains were used to detect calcification in paraffin sections [13]. These stages of histology were all carried out in the histological laboratory, Department of Pathological Analysis, Wasit University.

### Immunohistochemical technique

Standard primary antibody S100 calcium-binding protein B (S100B) was used with a DAB immune staining procedure to detect calcification in tissues, according to manufacturer’s instructions. First, the charged slides containing paraffin tissue sections were briefly dewaxed in absolute toluene. Slides were placed in decreasing concentrations of ethanol (absolute, 90% and 70%) and after washing 3 times were immersed in citrate buffer solution and heated in the oven to 100°C for antigen retrieval followed by cooling for 30 min at room temperature (RT) of 37°C. Slides were then washed 3 times with phosphate buffer saline (PBS) and incubated with protein blocking solution for 10 min at RT. Sections were treated with primary antibody for 30 min at RT, then rinsed with PBS 7 times. Slides were subsequently treated with one-step HRP polymer for 30 min at RT, then washed with PBS 7 times and distilled water (DW) 3 times. A few drops of DAB reagent were added to slides and left for at least 10 min at RT, then washed 7 times with PBS and then washed 7 times with DW. Sections were then immersed in hematoxylin staining solution for 1 min. Finally, stained slides were washed with DW until turning clear, mounted with DPX mounting medium, and enclosed with a coverslip.

### Statistical analysis

#### Digital image analysis

Slides were photographed with a photosystem including a photic microscope (Mejia) using 4×, 10×, 20×, and 40× objectives and prepared with a digital camera (Canon 20 megapixels). Final images were analyzed on an Intel Core I3 computer using VideoTest Morphology software (Russia), with a specific built-in routine for distance, % distance measurement, and required object counting.

For digital image analysis, all slides with von Kossa and Alizarin stain were initially pictured with 400× and adjusted to a final size of 12.7 cm in width and 9 in length, 300 dpi. In these images, 1 cm (vertical bar) is equal to 25 microns. These images were opened in ImageJ to determine reaction areas and calculate these as a percentage of the total image area. Comparisons were then made between images from different groups.

### Statistical analysis

One-way analysis of variance (ANOVA) test was used to compare more than 2 independent groups, with *post hoc* Tukey test used for pairwise comparison. Paired t-test was usedfor comparison between studied -groups. Repeated measures ANOVA test was used to compare more than 2 studied periods with *post hoc* Tukey test. p < 0.05 was considered statistically significant.

## Results

### Histopathology

**Figure-1:**
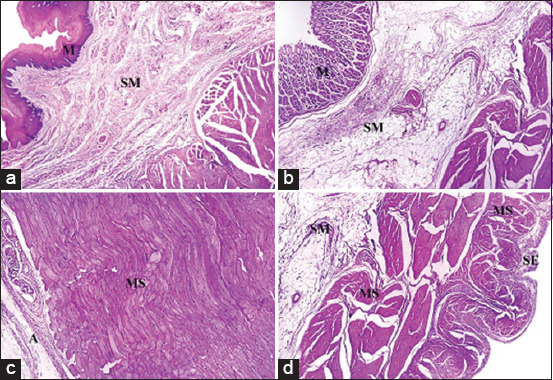
Photomicrograph showing a general histological structure of the esophagus and abomasum in a cow. (a) Mucosa (M); submucosa (SM) in the esophagus (hematoxylin and eosin [H&E] stain – 4×). (b) Mucosa (M); submucosa (SM) in the abomasum (H&E stain – 4×). (c) Tunica muscularis (MS); adventitia (A) in the esophagus (H&E stain – 4×). (d) Submucosa (SM); tunica muscularis (MS); serosa (S) in the abomasum. (H&E stain – 4×).

**Figure-2:**
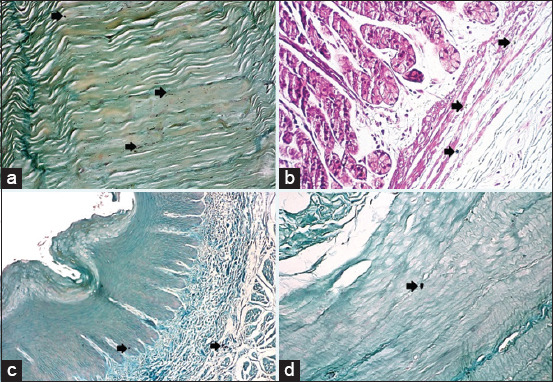
Photomicrograph showing: (a) The calcification (arrow) as dark fine points through the tunica muscularis of the esophagus of 1 year old of a cow (alizarin red – 20×); (b) the calcification (arrow) as fine points in the tunica submucosa of abomasum at 1 year old of the cow (von Kossa stain – 40×). (c) The calcification (arrow) as dark small points through the tunica mucosa and submucosa of the esophagus of 2 years old of a cow (alizarin red – 20×). (d) The calcification (arrow) as dark small points through the tunica muscularis of abomasum at 2 years old of a cow (alizarin red – 40×).

**Figure-3:**
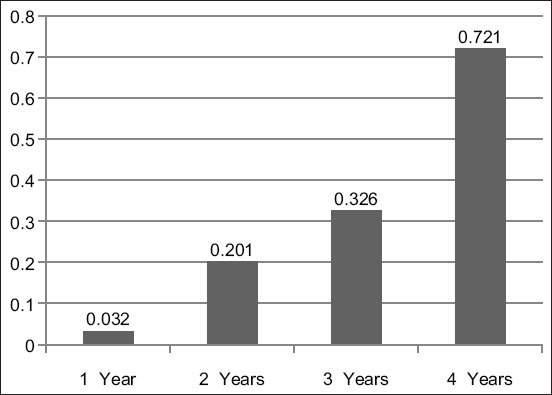
The relative calcification ratio of the esophagus and abomasum in cows of different ages (1 year old, 2 years old, 3 years old, and 4 years old).

**Figure-4:**
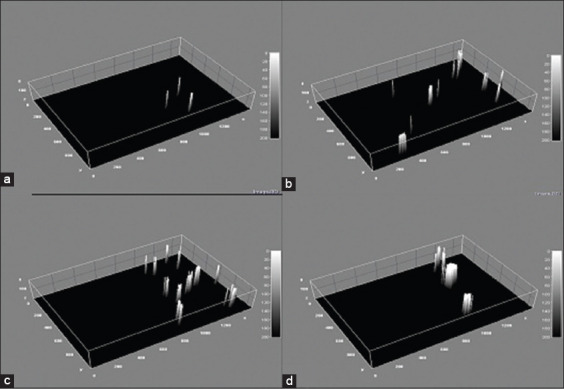
Statistical surface plot showing the percentage of calcification for the esophagus and abomasum of the cow: (a) At 1 year old. (b) At 2 years old. (c) At 3-year-old cow. (d) At 4 years old.

**Figure-5:**
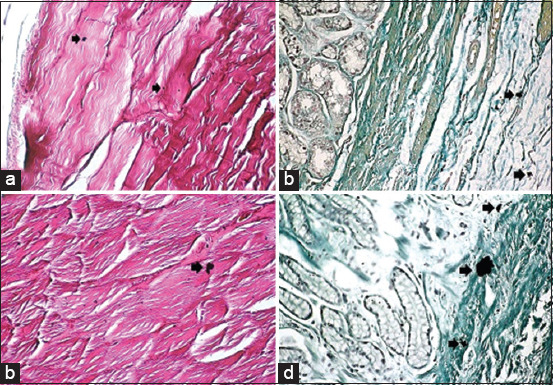
Photomicrograph showing: (a) The medium calcified spots (arrow) through the tunica muscularis of the esophagus at 3 years old of a cow (von Kossa stain – 20×). (b) The calcification (arrow) as medium spots in the tunica submucosa of abomasum at 3 years old of the cow (alizarin red – 40×). (c) The calcification (arrow) as large dark foci spread through tunica muscularis of the esophagus at 4 years old of a cow (von Kossa stain – 20×). (d) The calcification (arrow) as large dark foci through the mucosa and submucosa layer of abomasum at 4 years old of the cow (alizarin red – 40×).

Histological results from H&E stain showed the general structure of the cow esophagus and abomasum, which consist of four layers: Mucosa, submucosa, muscularis, and serosa, with the upper part of the esophagus also containing adventitia due to its location outside the abdominal cavity. Each layer contains a dominant type of tissue which contributes a specific function to digestion. In the esophagus, the mucosal layer features keratinized stratified squamous epithelium while the apex of the abomasum mucosal fold contains simple columnar epithelial tissue. The mucosa of both the esophagus and abomasum includes the lamina propria, which comprises connective tissue cells, fibers, and blood capillaries as well as few layers of smooth muscle forming the muscularis mucosae. In addition, the abomasal mucosa featured compound, coiled, and branched tubular glands in its cardiac region and straight, branched tubular glands in its neck, base, and body regions. Histological results showed distinct tunica submucosa in both the esophagus and abomasum, which contained loose irregular connective tissue interspersed with bundles of elastin, collagen, and a few reticular fibers along with small blood vessels (Figure-1a and b). In the cervical esophagus, the tunica muscularis consisted of a thick inner layer of striated skeletal muscle with a circular direction and a thinner outer layer with a longitudinal direction. In the abomasum, the tunica muscularis was comprised of smooth muscle fibers arranged in a circular inner layer and a longitudinal outer layer. Histological findings showed the cervical region of the esophagus covered by tunica adventitia, including loose irregular connective tissue with small blood vessels and fatty tissue. Meanwhile, the abomasum, because of its abdominal cavity location, contained tunica serosa possessing an outermost mesothelium layer along with loose connective tissue (Figure-1c and d). Microscopic examination of von Kossa and alizarin red staining in samples from 1-year-old cows revealed fine points of early calcification deposited in the tunica submucosa and tunica muscularis of both esophagus and abomasum (Figure-2a and b). The occurrence of calcification in the 1^st^ year was estimated as 0.032±0.0046 of the total esophagus or abomasum (Figure-3). In 2-year-old animals, small brown points of calcification were observed diffused in the mucosa, connective tissue of the submucosal layer, and muscle bundles of the muscular layer of esophagus and abomasum of all animals (Figure-2c and d). The occurrence of calcification at this age was estimated at 0.201±0.0081 (Figure-3), which was significantly (p ≤ 0.05) greater than in the 1-year-old group (Figure-4a and b). In 3-year-old animals, microscopic investigation revealed bulk increases in calcification, appearing as medium calcified spots spread throughout the submucosal and muscularis layers of both esophagus and abomasum (Figure-5a and b). The occurrence of calcification at this age was estimated at 0.326±0.0116 (Figure-3). Histological analysis of 4-year-old samples revealed obvious, advanced calcification, showing as large dark foci spread through deteriorating submucosal connective tissue and diffused among the skeletal muscle bundles of the muscularis layer in the esophagus and smooth muscle bundles of the tunica muscularis in the abomasum (Figure-5c and d). The esophagus and abomasum calcification morbidity at 4 years old was estimated at 0.721±0.0088 (Figure-3) and is compared with findings from other ages (Figure-4b,c and d).

### Immunohistochemical results

**Figure-6:**
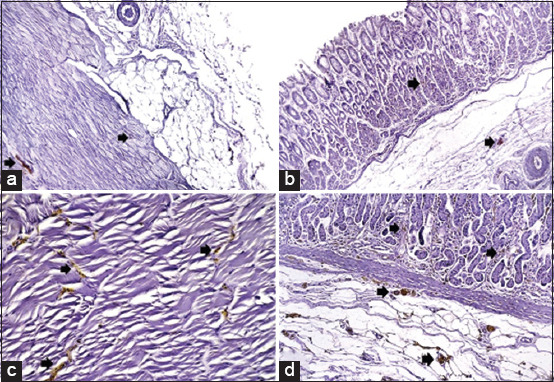
Photomicrograph showing: (a) The beginning of calcification as positive immunoreaction (arrow) in tunica muscularis, of the esophagus at 1-year-old cow (immunohistochemistry 10×). (b) The starting of calcification as positive immunoreaction (arrow) in tunica mucosa and submucosa of abomasum at 1-year-old cow (immunohistochemistry 20×). (c) The positive immunoreaction of calcification (arrow) in tunica muscularis of the esophagus at 2-year-old cow (immunohistochemistry 20×). (d) The positive immunoreaction of calcification (arrow) in tunica mucosa and submucosa of abomasum at 2-year-old cow (immunohistochemistry 20×).

**Figure-7:**
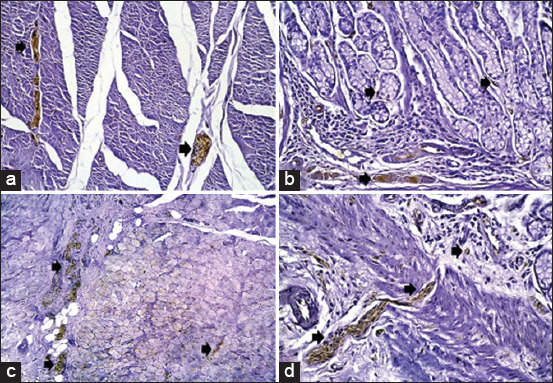
Photomicrograph showing: (a) The positive immunoreaction (arrow) of calcified areas in tunica muscularis of the esophagus at 3-year-old cow (immunohistochemistry 10×). (b) The positive immunoreaction (arrow) of calcified areas in tunica mucosa and submucosa of abomasum at 3-year-old cow (immunohistochemistry 40×). (c) The strong immunoreaction (arrow) of calcification in the muscularis layer of the esophagus at 4-year-old cow (immunohistochemistry 20×). (d) The strong immunoreaction (arrow) of calcification in tunica mucosa and submucosa of abomasum at 4-year-old cow (immunohistochemistry 40×).

Immunohistochemical examination using a primary antibody for calcium-binding protein S100B revealed calcification beginning in cows of 1 year old as a positive immunoreaction in the tunica submucosa and tunica muscularis. Furthermore, some calcification was also observed in the tunica mucosa of both the esophagus and abomasum of all cows of 1-year-old (Figure-6a and b). At 2 years old, the esophagus and abomasum sections showed positive immunoreactions for calcification, especially in the submucosa and muscularis layers, significantly (p ≤ 0.05) more than was observed in the previous age group (Figure-6c and d). At 3 years old, positive immunoreactions were increased in the calcified areas of the esophagus and abomasum sections appearing as medium spots (Figure-7a and b). At an advanced age of 4 years old, results showed strong immunoreactions at the site of calcium precipitation in the submucosa and muscularis layers of the esophagus and abomasum sections, which was higher than observed at previous ages (Figure-7c and d).

## Discussion

In the current study, marked spontaneous calcification was observed in the esophagus and abomasum of all animals, which progressed significantly with age. These pathological events were detected by histological technique and confirmed by immunohistochemistry. Examination of the GIT is extremely important in the detection of digestive diseases. Gastrointestinal symptoms, along with clinical pathologic correlation and repeated risks, can only be understood by careful clinical evaluation. The main histological characterizations of the esophagus and abomasum of cow in the present work were similar to those previously described in rabbit [14], in sheep [15], and in camel [16]. In the present study, our results revealed slight calcification in the mucosa, submucosa, and muscularis layers of esophagus and abomasum in young cows (1 year old), in keeping with the previous studies on the primary appearance of calcification in multiple organs, Masuda and Hirose [17] who mentioned the presence of numerous, point-like basic calcium phosphate calcifications over the articular surface, and soft tissues in mouse. Mild calcification of the heart valves has also been described in 1-year-old bulls [7]. On the other hand, one study [18] mentioned that soft-tissue calcification may be a result of a metastatic mechanism, leading to consequent systemic mineral imbalance which caused the underlying disease, such as hyperparathyroidism, or uremia, or by dystrophic mechanism. Another study [11] interpreted gastrointestinal calcifications as a benign process, especially in young individuals, and these calcifications are rarely recognized as malignant tumors. In the current study, immunohistochemistry revealed positive immunoreactions with the site of calcium precipitation in tunica submucosa and tunica muscularis in the esophagus and abomasum samples. These results were in agreement with the previous study [11] who observed intramural calcification within the wall of the colon. The present results also revealed slight positive immunoreaction with the areas of calcification in the mucosal layer in both digestive organs. This was in keeping with a previous study [19] demonstrating that gastric mucosal calcification is a very rare pathology which may develop secondary to numerous diseases. Calcium precipitation in the mucosal layer of these tissues is probably due to the secretion of mucous glands. Mucin-producing cells create a relatively alkaline environment [20], which is conducive to the deposition of calcium salts, especially phosphate and carbonate salts [21]. Several authors have classified gastric mucosal calcinosis as a metastatic calcification subtype, defined as the precipitate of calcium salt in normal soft tissue [22]. Recently observed, metastatic calcification is a common subtype of calcification that occurs due to disorders in serum biochemical environments, such as hypercalcemia and hyperphosphatemia [13]. In this study, histological results affirmed by immunohistochemical findings showed calcification in the esophagus and abomasum progressed with advancing age, eventually resulting in large spots of calcium deposit by 4 years old. These findings were similar to a previous description [23] of mineralization throughout stomach sections of aged rodents, characterized by focal aggregates of densely staining mineral in gastric tunica muscularis, and a band running parallel to the mucosa. This gastric mineralization may become obvious when there is a metabolic mineral disturbance, like such as calcium-phosphorus metabolism or hyperparathyroidism. However, calcification in the wall layers of some parts of the GIT has never been reported in domesticated animals. Results from our study are necessarily associated with physiological disturbance in the blood calcium and phosphate or pathological condition such as chronic kidney disease or tumor, which can be concomitant in animals without clinical signs. Some previous studies have claimed that several gastrointestinal tumors may also contain calcification [24]. Moreover, some mechanisms of calcification in tumors have been suggested [25]: (a) Calcium salts are deposited within the tumor as an outcome of a secretory function of the carcinoma, (b) dystrophic calcification happens within sites of tumor necrosis, and (c) metastatic calcification occurs as a consequence of hypercalcemia. Furthermore, some researchers have reported that dystrophic or metastatic calcification of the stomach may be secondary to hypercalcemia, hypervitaminosis A, hyperphosphatemia, chronic kidney disease, gastric neoplasia, and tumor lysis syndrome or may be associated with receiving aluminum-containing antacids, sucralfate or bismuth, or citrate-containing blood products [26]. Most gastric calcifications rarely cause symptoms such as dyspepsia or epigastric pain [21].

## Conclusion

This study revealed the occurrence of esophageal and abomasum calcification in different ages of a cow. Spontaneous calcifications appeared as mild spots in epithelial, connective, and muscular layers of these tissues at a young age. Prominently, the pattern of calcification significantly increased with age. These pathological events may be associated with a wide spectrum of physiological disturbances which result in abnormal blood biochemical environment, such as hypercalcemia and hyperphosphatemia, or secondary to other clinical conditions.

## Authors’ Contributions

BAA and EAKZ projected and directed the plan. BAA and AMA made the practical parameters in the immunohistochemical laboratory. AMA and EAKZ adopted the pictures and formal analysis. AMA composed the primary exemplar of the article. EAKZ and AMAB drafted and revised the manuscript. All authors read and approved the final manuscript.
